# Iterative Bayesian Estimation of Travel Times on Urban Arterials: Fusing Loop Detector and Probe Vehicle Data

**DOI:** 10.1371/journal.pone.0158123

**Published:** 2016-06-30

**Authors:** Kai Liu, Meng-Ying Cui, Peng Cao, Jiang-Bo Wang

**Affiliations:** 1 School of Transportation and Logistics, Dalian University of Technology, Dalian, 116024, P.R. China; 2 College of Science and Engineering, University of Minnesota, Twin Cities, Minneapolis, MN, 55455, United States of America; 3 National United Engineering Laboratory of Integrated and Intelligent Transportation, School of Transportation and Logistics, Southwest Jiaotong University, Chengdu, 610031, P.R. China; Beihang University, CHINA

## Abstract

On urban arterials, travel time estimation is challenging especially from various data sources. Typically, fusing loop detector data and probe vehicle data to estimate travel time is a troublesome issue while considering the data issue of uncertain, imprecise and even conflicting. In this paper, we propose an improved data fusing methodology for link travel time estimation. Link travel times are simultaneously pre-estimated using loop detector data and probe vehicle data, based on which Bayesian fusion is then applied to fuse the estimated travel times. Next, Iterative Bayesian estimation is proposed to improve Bayesian fusion by incorporating two strategies: 1) substitution strategy which replaces the lower accurate travel time estimation from one sensor with the current fused travel time; and 2) specially-designed conditions for convergence which restrict the estimated travel time in a reasonable range. The estimation results show that, the proposed method outperforms probe vehicle data based method, loop detector based method and single Bayesian fusion, and the mean absolute percentage error is reduced to 4.8%. Additionally, iterative Bayesian estimation performs better for lighter traffic flows when the variability of travel time is practically higher than other periods.

## Introduction

Travel time, which is well understood by both traffic engineers and the public, is one of the most important measures for accessing the operating efficiency of a traffic system. Travel time estimation in urban arterials is a challenging subject because urban traffic flow is affected by lots of uncertain factors (driving behaviours, weather conditions, incidents, etc.) and interrupted periodically by traffic signals [1–4].

Traditionally, Loop detector data (e.g. volume, speed or occupancy) are widely used to estimate urban link travel times [5–9]. As a mature traffic data collection method, loop detectors continuously provide relatively accurate and reliable traffic information on fixed points of roads. Recent years estimating travel time from probe vehicle data has attracted increasing attentions of researchers [9–12]. A major reason is that single vehicle travel time can be directly calculated from probe data with higher polling frequency. However, subject to the low penetration ratio, low polling frequency and limited types of probe vehicles, it is still difficult to accurately estimate arterial travel time from the single type of probe vehicle data [13, 14]. Based on these facts, data fusion becomes a more realistic direction for estimating accurate and reliable travel time on urban arterials [15–18].

The traffic condition estimated from individual sensors may be incomplete, inconsistent or/and imprecise. Additional data sources may provide complementary information on traffic, and fusion of different information is potential to produce a better understanding of the actual traffic condition, by decreasing the uncertainty related to the single source [18].

The objective of this research is to provide a practical fusing method with higher reliability for traffic condition estimation. This paper attempts to address some practical issues in data fusion: 1) information from both loop data and probe data may be uncertain, imprecise and even conflicting, 2) neither loop detector data nor probe data is stable in performance, and 3) information about which data performs better is unavailable.

The remaining of this paper is organized as follows. Chapter 2 reviews the widely used fusion methods and sums up some unresolved issues. Chapter 3 details the proposed methodology, followed by the performance evaluation with data derived from VISSIM simulation in Chapter 4. In the final section, some conclusions and future research are discussed.

## Literature Review

Travel time estimation is a popular topic and has been studied for many years. In this section, we make an in-depth review on researches mostly related to this paper. A systematic review can be referred in [19].

The objective of data fusion in the field of advanced transportation information system (ATIS) is to develop better performed estimation on a system from kinds of independent data sources. In order to provide more reliable traffic information efficiently, fusing the stationary sensor data and mobile probe data is perceived as a well-adapted choice for satisfying the operational needs of traffic operators and traffic information centres [20].

The accuracy of fused link travel time had been proved to be superior to single loop detector data and probe vehicle data, Choi and Chung introduced an algorithm in detail on the probability inference for fusing loop detector data and probe vehicle data [21], their milestone work put forward a lot of successful researches with various focus on methodology, objectives and applications.

An optimal fusion method would be able to make full use of the useful information from these two types of data sources [20]. It is well known that there is a strong complementarity between these two types of data. On the one hand, continuous loop detector data can make up for the lack of probe data in time intervals when probe vehicles are not observed. On the other hand, wide coverage probe data can provide traffic information for areas where loop detectors are not installed.

Data fusion approaches in link travel time estimation are proposed for various types of data where different uncertainty may be observed. Some fusion techniques for handing biases of various kinds are developed, through simulation [9] or experimental research [15, 20–23].

Among all the fusion techniques, three mainly groups are widely used, namely statistical models (such like weighted mean, Bayes fusion, and so on), the analytical model (such like evidence theory), and data-driven model (such like learning machine, Fuzzy logic, artificial neural networks). However, there is no evidence that if the data source is incomplete, inconsistent or/and imprecise, none of above methods could deal with the travel time estimation of arterial links with signal intersections very well. For example, when using the method of weighted mean, actually various data active differently during different situations due to unstable errors and uncertainties, the weights, reflection of reliability of data source, could not be pre-determined. Thus, Choi and Chung stated that the fusion algorithm should be tested and calibrated for different traffic conditions [21].

Moreover, traditionally data fusion methods deal with the objective of yielding a single probability distribution of link travel times, from which more reliable inference can be made, while the travel time distribution of links in urban network with intersections is actually multimodal distribution, rather than any kind of unimodal distribution, due to the signal control at intersections and diversities in following speeds among groups with different driving habits. Furthermore, the common statistical period for link travel time estimate, such as 5 minutes in most cases, cannot always cover several whole signal cycles, and therefore leads to great observation bias, which contributes significantly to the estimation errors if considering the time sequence and do harm to the later step of data fusion [24].

Zheng and Van Zuylen stated that in different traffic condition the arrival patterns lead to absolute different influence on delays distribution at signal intersections [25], because the uncertainty induced by both traffic conditions and traffic control at intersections become more complicated. Based on the kinematic wave theory and variational theory, Mehran et al. proposed a method to obtain more accurate link travel times by reconstructing vehicle trajectories combining traffic data from fixed and probe detectors according to traffic flow principles [26]. However, relative higher polling frequency of probe data is required to reproduce the time-space trajectory, which is not always available in practice [13]. The mechanism of dealing with uncertainty in link travel time estimation is not well addressed in previous studies. There is a gap between understanding the travel time uncertainty and the estimation method which need to be self-adaption from various data.

## Methodology

### Travel time estimation from loop detector data

Single or dual loop detectors continuously detect the passing of vehicles through a short segment of a road. Loop detector data include volume, speed and occupancy. Many models have been presented to estimate travel times based on loop detector data. Although some integrated Bayesian models [27, 28] may improve estimation, these statistical models cannot always dynamically measure the variability of delay at signalized intersections [25]. In the latest Highway Capacity Manual [8], a practical methodology is presented to estimate travel times on urban streets. We briefly summarise this method as follows and employ it in this study.

We denote the travel time derived from loop detector data by *t*_*loop*_. The travel time on a urban road link consists of two components:

$$t_{loop}=t_{f}+d_{c}$$ (1)

where *t*_*f*_ is the average free-flow travel time, *d*_*c*_ is the control delay associated with vehicles slowing in advance of an intersection.

Assuming that *s*_*f*_, average instantaneous speed, was the average running speed for the link, then *t*_*f*_ could be expressed as:

$$t_{f}=L/s_{f}$$ (2)

where *L* is the length of the link.

Assuming no lanes has an initial queue on the analysis road, then the control delay is computed as [8]:

$$d_{c}=d_{1}+d_{2}$$ (3)

$$d_{1}=\frac{0.5C{\left(1-\frac{g}{C}\right)}^{2}}{1-\left[\min\left(1,X\right)g/C\right]}$$ (4)

$$d_{2}=900\tau \left(X-1\right)+\sqrt{{\left(X-1\right)}^{2}+\frac{8\gamma \alpha X}{c\tau }}$$ (5)

with *X* = *q*/*c*

where

*d*_1_ = uniform control delay,

*d*_2_ = incremental delay,

*C* = cycle length (s),

*g* = effective green time (s),

*X* = volume-to-capacity ratio,

*τ* = analysis time period (s),

*c* = capacity (veh/h),

*q* = demand flow rate,

*γ* = incremental delay factor, and

*α* = upstream filtering adjustment factor.

### Travel time estimation from probe vehicle data

Probe vehicle, also known as floating car, is a vehicle equipped a recording device receiving signals from GPS or antenna (GPS-based or cellular phone-based) and can provide series of points with information time stamp, speed and location etc. along the vehicle trajectories [11, 29]. In general, estimating link travel time from probe vehicle data involves at least two steps: 1) decomposing travel times measured by probe vehicle into individual links; 2) estimating link travel time from the decomposed link travel times of probe vehicles [9, 30, 31]. However, the accuracy of decomposed link travel times is unstable because of randomly stop-and-go behaviors of vehicles and GPS data deviation in urban roads [13]. Sanwal and Walrand proposed an improved travel time estimate by applying an adaptive exponential smoothing (AES) method [23].

We denote the estimated link travel time from probe vehicle data in the kth time interval by *t*_*probe*_(*k*). Assume that *n*(*k*) probe vehicles are observed in the same time interval, and the decomposed link travel time of the m^th^ probe vehicle is *t*^*m*^, then *t*_*probe*_(*k*) can be calculated as a weighted average:

$$t_{probe}\left(k\right)=t_{probe}\left(k-1\right)+\alpha \left(k\right)\left\{\left[\frac{1}{n\left(k\right)}\sum_{m=1}^{n\left(k\right)}t^{m}\right]-t_{probe}\left(k-1\right)\right\}$$ (6)

where *α*(*k*) represents the dependence of link travel time on time, *α*(*k*) is determined by minimizing the mean squared estimation error *e*_*k*_.

Assume the estimation error *e*_*k*_ is normally distributed with mean of 0, *α*(*k*) can be derived as

$$\alpha \left(k\right)=\frac{n\left(k\right)}{1+n\left(k\right)}\cdot \left|\frac{E_{k}}{A_{k}}\right|$$ (7)

where *E*_*k*_ is the smoothing error, and *A*_*k*_ is the absolute smoothing error.

*E*_*k*_ and *A*_*k*_ follow the equations:

$$E_{k}=r\cdot e_{k}+\left(1-r\right)\cdot E_{k-1}$$ (8)

$$A_{k}=r\cdot \left|e_{k}\right|+\left(1-r\right)\cdot A_{k-1}$$ (9)

where *r* is the weight coefficient.

$$r=\frac{n\left(k\right)}{n\left(k\right)+1}$$ (10)

$$e_{k}=\left[\frac{1}{n\left(k\right)}\sum_{m=1}^{n\left(k\right)}t^{m}\right]-t_{probe}\left(k-1\right)$$ (11)

In AES method, the continuity of traffic flow is considered in term of the dependence of link travel time on time. It should be note that, for time interval when no probe vehicle is observed, i.e. *n*(*k*) = 0, the estimated travel time $\hat{t}(k)$ equals to the estimate on previous time interval $\hat{t}(k-1)$. This feature of AES method can guarantee the continuity of estimates among adjacent time intervals.

### Bayesian fusion

The Bayesian fusion applies the theory of probabilities with the Bayesian framework. Within this approach, the source/sensor evidence is represented probabilistically and Bayes’ rule is used to perform fusion process [32], which could improve the estimation when data are uncertain, imprecise and conflicting [33]. In our problem, two sensors are loop detector and probe vehicle, respectively. With the measured data set {*t*_*loop*_, *t*_*probe*_}, the probability of fused link travel time *μ* conditioned on the measured data set can be given as:

$$P\left(\mu |t_{loop},t_{probe}\right)=\frac{P\left(\mu ,t_{loop},t_{probe}\right)}{P\left(t_{loop},t_{probe}\right)}$$ (12)

Assume that *μ*, *t*_*loop*_ and *t*_*probe*_ follow normal distributions $N(\mu_{0},\sigma_{0}^{2})$, $N(\mu ,\sigma_{loop}^{2})$ and $N(\mu ,\sigma_{probe}^{2})$, respectively. And then *P*(*μ*|*t*_*loop*_, *t*_*probe*_) can be given as:

$$P\left(\mu |t_{loop},t_{probe}\right)=\beta exp\left\{-\frac{1}{2}\left[{\left(\frac{t_{loop}-\mu }{\sigma_{loop}}\right)}^{2}+{\left(\frac{t_{probe}-\mu }{\sigma_{probe}}\right)}^{2}+{\left(\frac{\mu -\mu_{0}}{\sigma_{0}}\right)}^{2}\right]\right\}\times \frac{1}{2\pi \sigma_{loop}\sigma_{probe}}$$ (13)

As we can see, the exponential part of *P*(*μ*|*t*_*loop*_, *t*_*probe*_) is a quadratic function of *μ*, thus *μ*|*t*_*loop*_, *t*_*probe*_ also follows a normal distribution represented by $N(\mu_{N},\sigma_{N}^{2})$. And then *P*(*μ*|*t*_*loop*_, *t*_*probe*_) can also be given as:

$$P\left(\mu |t_{loop},t_{probe}\right)=\frac{1}{\sqrt{2\pi }\sigma_{N}}exp\left\{-\frac{1}{2}{\left(\frac{\mu -\mu_{N}}{\sigma_{N}}\right)}^{2}\right\}$$ (14)

A comparison of the parameters of formulas (13), (14) shows that:

$$\mu_{N}=\frac{\frac{t_{loop}}{\sigma_{loop}^{2}}+\frac{t_{probe}}{\sigma_{probe}^{2}}+\frac{\mu_{0}}{\sigma_{0}^{2}}}{\frac{1}{\sigma_{loop}^{2}}+\frac{1}{\sigma_{probe}^{2}}+\frac{1}{\sigma_{0}^{2}}}$$ (15)

Finally, the Bayesian estimation of *μ* is calculated as:

$$\hat{\mu }=\int_{\Omega }^{}\mu P\left(\mu |t_{loop},t_{probe}\right)d\mu =\mu_{N}$$ (16)

Thus *μ*_*N*_ can be considered as the Bayesian estimation of *μ*. And we use *μ*_*N*_ to represent $\hat{\mu }$ in subsequent contents.

### Iterative estimation

**Fig 1:**
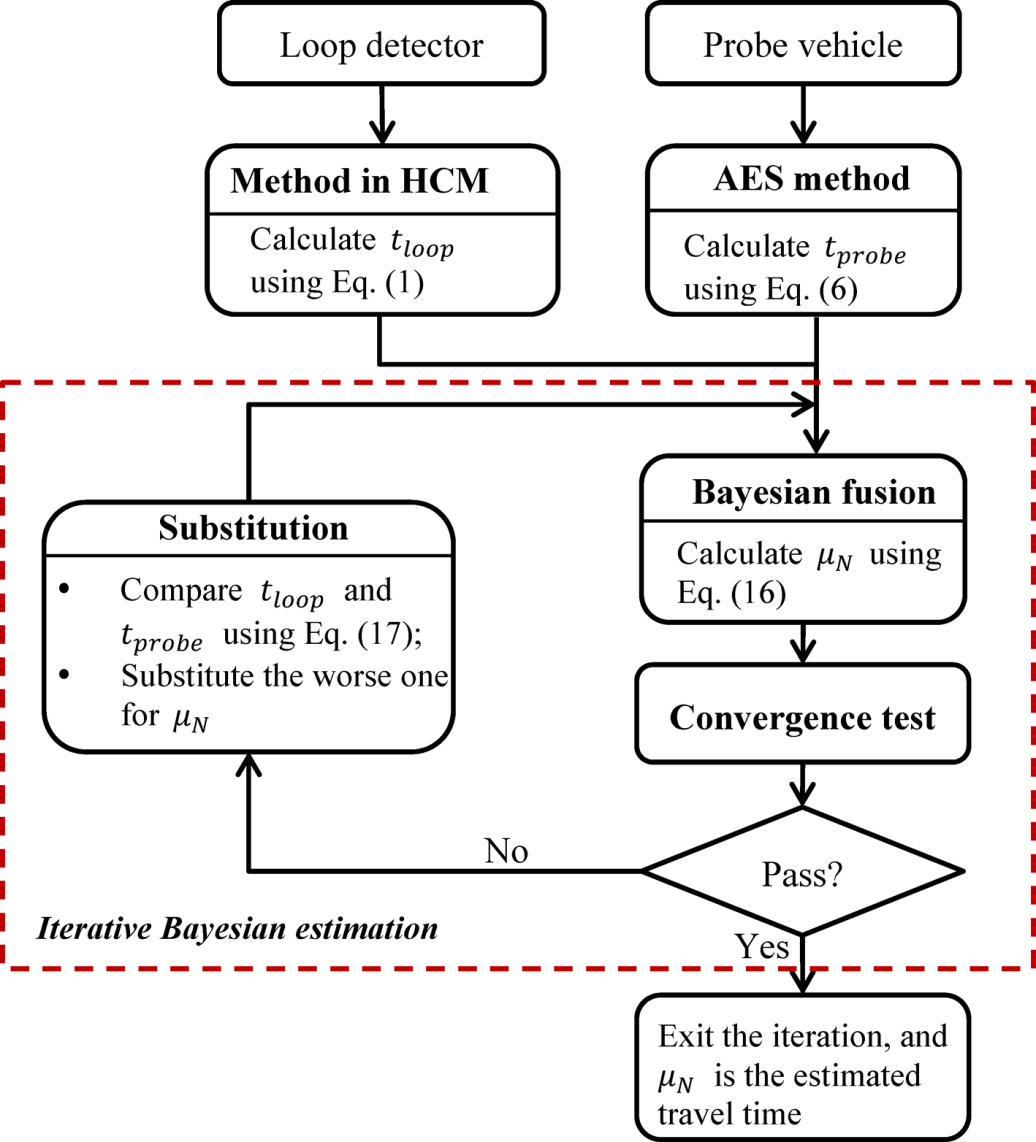
Flow chart of the iterative Bayesian estimation.

In practice data sources might provide uncertain or imprecise information. For instance, sometimes there are few probe vehicles observed on a link of interest during certain time interval. In this case, travel time estimated from probe vehicle data is probably biased, and fusing this information would possibly not only fail to improve the estimate, but reduce the estimation accuracy. Therefore, we develop an iterative estimation scheme to improve Bayesian fusion. The basic idea is that, the Bayesian fusion is implemented iteratively, and at each iteration the fused link travel time is substituted for the worse estimation until conditions for convergence are satisfied. The proposed scheme is called iterative Bayesian estimation, depicted in Fig 1.

The iterative Bayesian estimation consists of three main parts: Bayesian fusion, substitution and convergence test. The former one is the core, and it can also be regarded as normal Bayesian estimation when iteration is 0, while the latter two are crucial for the estimation accuracy.

#### Substitution

**Fig 2:**
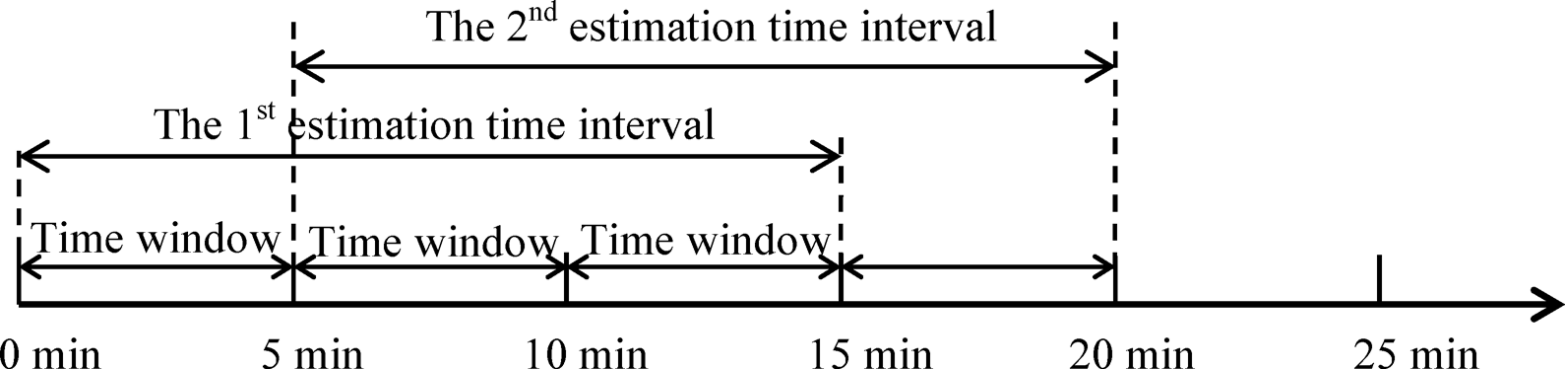
An example of estimation time interval and time window.

The substitution strategy aims to improve the Bayesian fusion through replace the lowest accurate travel time measured by one sensor with the current fused travel time. However, it is difficult to access the real accuracy of measured travel time, since the real travel time is unknown. For this reason, we develop an alternative method of accuracy measurement from two aspects. First, travel times estimated from historical probe vehicle data using Eq (6) are treated as the reference. In particular, probe vehicle data are accumulated in the same time interval of a day from the last two months, with separated workday and weekend. This treatment is based on the fact that there is high similarity for traffic patterns in the same time of similar days for a period of time. Second, the estimation time interval is divided into several identical time windows, and then the calculation of travel time evolves with time windows. For instance, if the estimation time interval is 15 mins and time window is 5 mins, and then the calculation of reference and measured travel time would be conducted every 5 mins for the estimation time interval from 0 to 15 mins (see Fig 2).

Suppose *t*_*ij*_ is the measured travel time from the *i*^*th*^ sensor in the *j*^*th*^ time window, and ${\tilde{t}}_{j}$ is the reference travel time from historical probe vehicle data in the *j*^*th*^ time window, *k* is the number of sensors, *m* is the number of time windows in one estimation time interval, then we use Euclidean distance to access the accuracy of measured travel time:

$$D_{i}=\sqrt{\sum_{j}^{m}{\left(t_{ij}-{\tilde{t}}_{j}\right)}^{2}}\;for\;i\;equals\;1\;to\;k$$ (17)

Therefore, the measured travel time with the maximum Euclidean distance to the reference travel time is of the lowest accuracy, and will be substituted for the current fused travel time in Bayesian fusion at next iteration.

#### Convergence test

**Fig 3:**
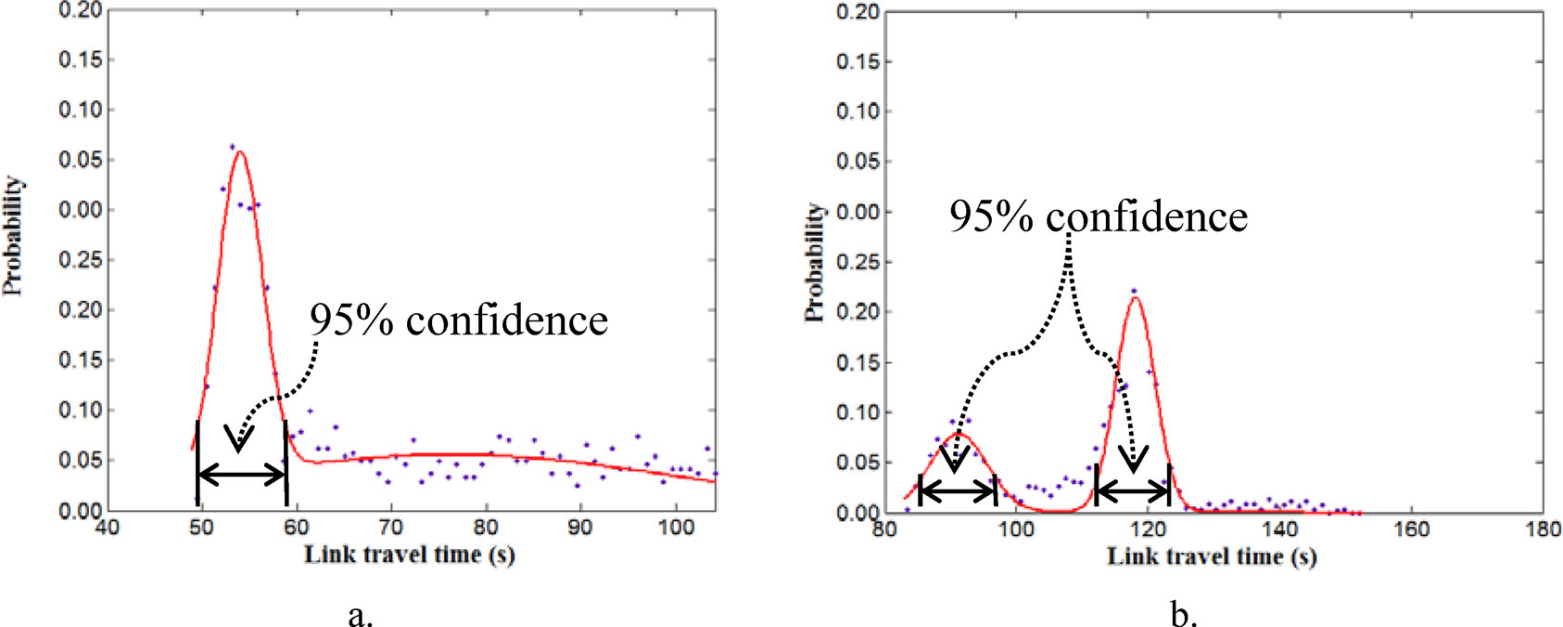
Empirical distributions of link travel time. a. An unimodal travel time distribution; b. An bimodal travel time distribution.

Conditions for convergence not only determine the termination of iterative program, but also are significant for the accuracy of the final estimation results. In order to get reasonable estimates, we define a condition for convergence based on travel time distribution from historical probe vehicle data. Generally, there are two typical kinds of link travel time distribution for urban arterials: unimodal (see Fig 3(A)) and bimodal (see Fig 3(B)). Data of Fig 3 are accumulated in 5-mimute interval from probe vehicle data in 30 days. In Fig 3(A), the unique peak occurs when link travel time is around 120 seconds. Whereas, in Fig 3(B), one peak occurs around 92 seconds and the other peak occurs around 117 seconds. These figures represent constant patterns of travel time distribution in two different links in an urban network. Thus, the estimated travel time would also follow the historical traffic pattern as long as no incident occurs. Therefore, the reasonable ranges of travel time can be determined by setting confidence level be 95%, based on the fitted curve from historical probe data (red curve in Fig 3).

If the fused travel time fall into the defined range, exit the iteration, and the fused travel time is the estimated travel time. If not, make substitution and enter the next iteration. However, this way would probably encounter a problem of endless loop. This problem occurs when the fused travel time is of very low accuracy because of few probe vehicles observed. For this reason, we add a second condition for convergence: relative error between fused times at two sequent iterations. If it is less than 0.1, the travel time with the highest probability in historical distribution as in Fig 3 is used as the final estimation result, and then the program terminates. The reason of this treatment is that, when the fused travel time is out of the reasonable range, historical information would be more credible than the fused travel time.

## Case Study

**Fig 4:**
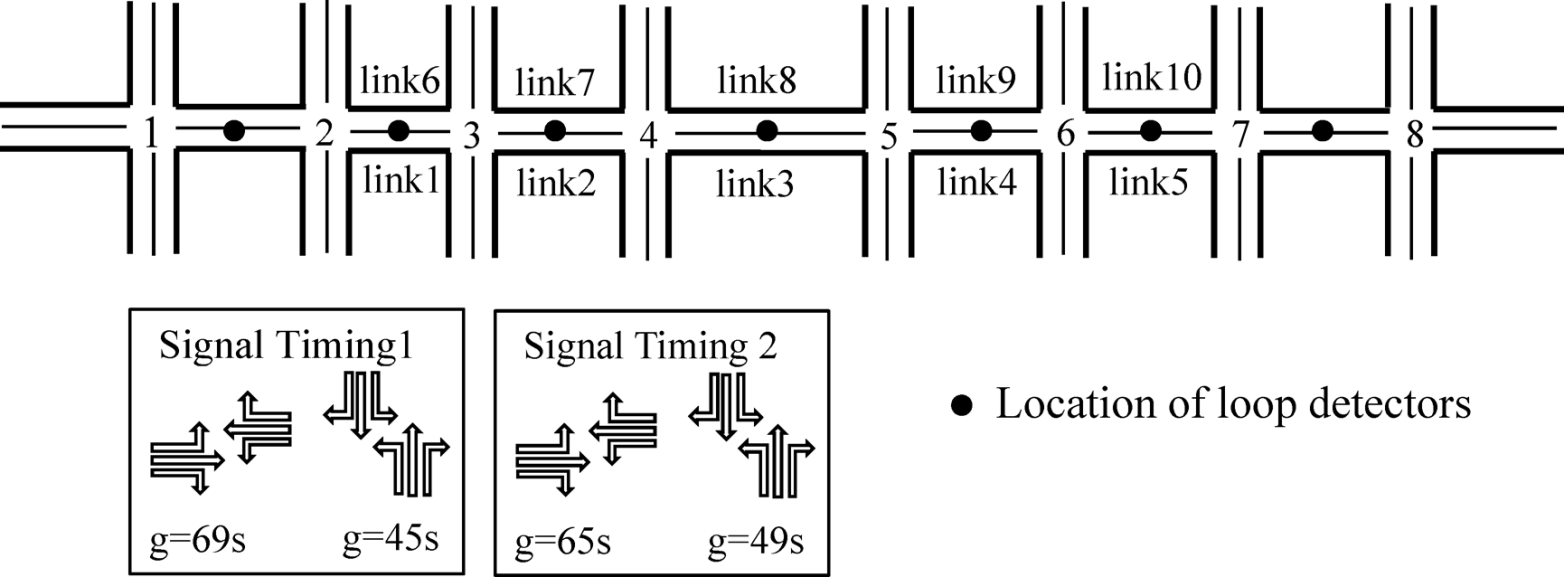
Study network.

We develop a test network in VISSIM to access the performances of the iteration Bayesian Estimation. The test network consists of 18 links and 8 intersections as in Fig 4. To simulate the real urban arterials, we choose 10 links in middle of this network as study links. The length of study links range from 600 m to 1000 m. Traffic flow on all links is controlled by traffic signals with cycle time of 120s, and signal timings are shown in Fig 4, where the intersection 1, 2, 7 and 8 use signal timing 1 and the intersection 3, 4, 5 and 6 use signal timing 2. The penetration rate of probe vehicles is assumed to be 5%, and a probe vehicle records its position every 30s. According to our preliminary experiments, installing loop detectors in the middle of links would provide better information for travel time estimation [34]. Therefore, every loop detector is set in the middle of each link. The total simulation time is 3 hours and 10 minutes from 6:50–10:00 am. The study time of 7:00–10:00 am is separated into three intervals depending on the input flow rates from entrance arterial and branch separately: before peak hour with 800pcu/h and 500pcu/h; peak hour with 1500pcu/h and 800pcu/h; after peak hour with 1000pcu/h and 600pcu/h.

Link travel time is estimated for each link in each time interval of 5 minutes, and the confidence level is set 95%. In order to access the estimation accuracy, we calculate the mean absolute percentage error (MAPE) for each link as:

$$MAPE=\frac{1}{N}\sum_{i=1}^{N}\frac{\left|{\hat{T}}_{i}-T_{i}\right|}{T_{i}}$$ (18)

where ${\hat{T}}_{i}$, *T*_*i*_ are the estimated and true travel time for the *i*^*th*^ time interval, respectively, and *N* is the number of estimation time interval.

**Fig 5:**
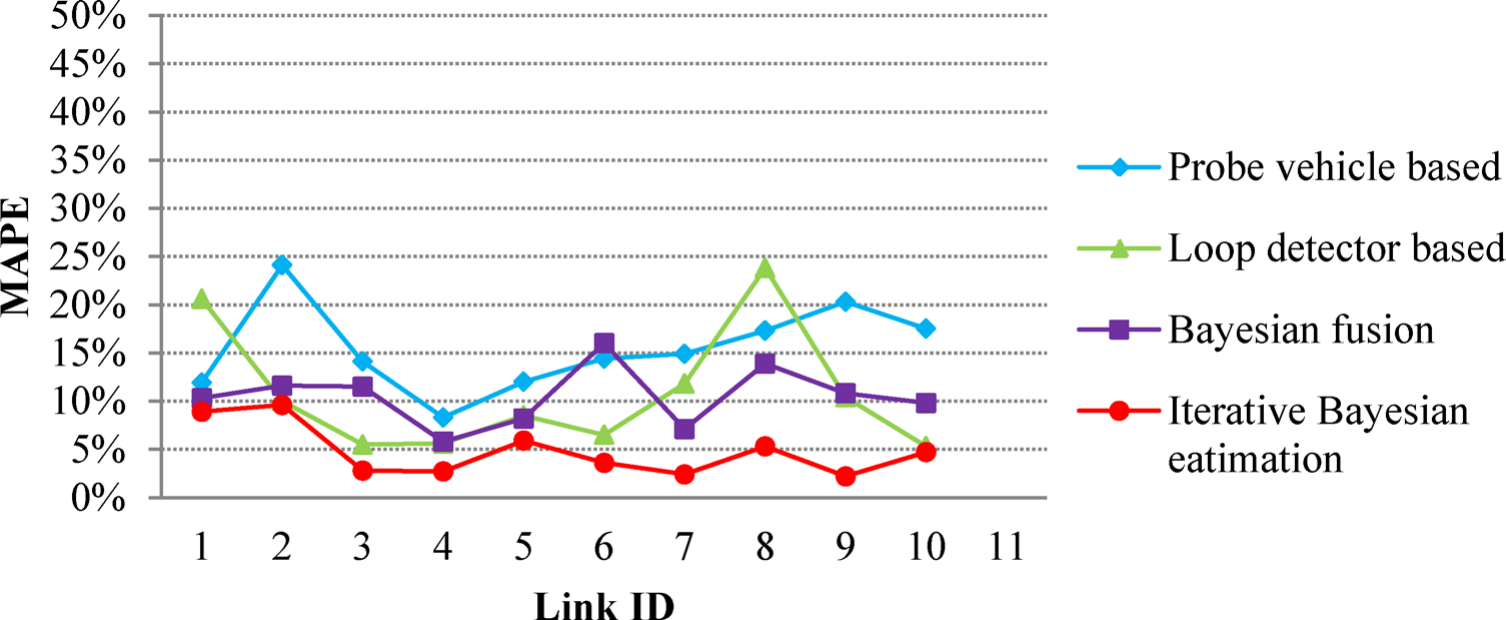
Comparison of various travel time estimation methods.

Lower value of MAPE means higher estimation accuracy. Link travel times are estimated using four methods presented in the section of methodology: probe vehicle based, loop detector based, Bayesian fusion, and iterative Bayesian fusion. The MAPEs of the estimated link travel times are calculated and demonstrated in Fig 5.

As we can see, the MAPEs of iteration Bayesian estimation for all links are the smallest among all four methods. This indicates that iteration Bayesian estimation outperforms the other three methods. We can also see, Bayesian fusion doesn’t always perform better than probe vehicle based method and loop detector based method, indeed it makes a certain amount of compromise between the latter two. This conforms that Bayesian fusion is capable to use the complementarity characteristic of two source data, but is unable to treat source data with various accuracies differently. This shortage of Bayesian fusion is covered by the proposed iteration scheme. Another observation is that the MAPEs of probe vehicle based method are larger than that of loop detector based method for most links, which means that 5% of probe vehicles might include much more uncertain information than loop detectors. Last but not least are the improvement of iteration Bayesian estimation comparing to the other three methods. The average MAPEs of various methods are: 15.5% for probe vehicle based; 10.8% for loop detector based; 10.5% for Bayesian fusion; and 4.8% for iteration Bayesian estimation. This means that iteration Bayesian estimation improves the travel time estimation accuracy by 10.7% compared with probe vehicle based method; 6.0% compared with loop detector based method; 5.7% compared with Bayesian fusion.

In order to analyse the effect of confidence level (CF) on the estimation accuracy, we implement iteration Bayesian estimation by setting three confidence levels: 95%, 90% and 85%, and calculate the MAPEs for each link (Table 1). It is observed that CF indeed have impacts on the estimation accuracy, with the MAPEs for each link increase with lower CF. However, it doesn’t mean that the higher CF the result is better, since higher CF means larger reasonable range for the fused travel time and consequently worse fused travel time will be accepted as the estimate travel time.

**Table 1:** Values of MAPE for various confidence levels.

Link ID	1	2	3	4	5	6	7	8	9	10	Average
CF = 95%	8.9	9.6	2.8	2.7	5.9	3.6	2.4	5.3	2.2	4.7	4.8
CF = 90%	9.3	11.6	2.8	3.0	5.9	3.6	3.1	8.4	2.7	4.7	5.5
CF = 85%	10.3	12.0	3.1	4.1	5.9	3.6	3.7	10.3	4.0	4.7	6.2

We also calculate the average MAPE of all links for various time periods with different traffic conditions (Table 2). As we can see, the MAPE value is the smallest for traffic condition before peak hour and the largest for that during peak hour. That is to say, iterative Bayesian estimation performs better for lighter traffic flows when the variability of travel time is practically higher than other periods.

**Table 2:** Average MAPE of all links for various traffic flow rates.

Time period	Before peak hour	Peak hour	After peak hour
**MAPE (%)**	2.9	5.9	3.6

## Conclusions

Fusing loop detector data and probe vehicle data to estimate link travel time is one important issue considering the uncertain, imprecise and even conflicting in single data source. Up to now, there is not much research having solved this issue well. The difficulty lies in that, on one hand point-based loop detector data doesn’t directly include information of travel time, on the other hand space-based probe vehicle data is of low penetration and polling frequency. In this paper, an iterative Bayesian estimation method is proposed to estimate link travel time from both loop detector and probe vehicle data. The results show that the proposed method outperforms probe vehicle data based method, loop detector based method and single Bayesian fusion, and the mean absolute percentage error is reduced to 4.8%. This improvement benefits from the substitution strategy, the moving estimation time intervals and the design of conditions for convergence in the iterative scheme, which take into account the information from historical probe vehicle data. The proposed method can also give a reasonable estimate even if there are few probe vehicles observed.

Although the proposed method of iterative Bayesian estimation performs well in the test simulation network, the actual performance should be evaluated in real network where traffic is much more complex. The travel time distribution estimated from historical probe vehicle data is applied in the proposed method. If traffic pattern changes, the credibility of the historical travel time distribution will decrease, and consequently the estimation accuracy will reduce. Future studies should address these issues.

## Supporting Information

S1 Dataset Vehicle Record from Day 1 to Day 4. (RAR)
